# Bio‐Based Solar Energy Harvesting for Onsite Mobile Optical Temperature Sensing in Smart Cities

**DOI:** 10.1002/advs.202104801

**Published:** 2022-03-28

**Authors:** Sandra F.H. Correia, Ana R.N. Bastos, Margarida Martins, Inês P.E. Macário, Telma Veloso, Joana L. Pereira, João A.P. Coutinho, Sónia P.M. Ventura, Paulo S. André, Rute A.S. Ferreira

**Affiliations:** ^1^ Department of Physics, CICECO – Aveiro Institute of Materials University of Aveiro Aveiro 3810‐193 Portugal; ^2^ Instituto de Telecomunicações and University of Aveiro Campus Universitário de Santiago Aveiro 3810‐193 Portugal; ^3^ Department of Chemistry, CICECO – Aveiro Institute of Materials University of Aveiro Aveiro 3810‐193 Portugal; ^4^ Department of Biology, CESAM University of Aveiro Aveiro 3810‐193 Portugal; ^5^ Department of Electrical and Computer Engineering, Instituto de Telecomunicações Instituto Superior Técnico Universidade de Lisboa Lisbon 1049‐001 Portugal

**Keywords:** Internet of Things, luminescent solar concentrator, nature‐based, self‐power, solar, temperature sensor, zero energy buildings

## Abstract

The Internet of Things (IoT) fosters the development of smart city systems for sustainable living and increases comfort for people. One of the current challenges for sustainable buildings is the optimization of energy management. Temperature monitoring in buildings is of prime importance, as heating account for a great part of the total energy consumption. Here, a solar optical temperature sensor is presented with a thermal sensitivity of up to 1.23% °C^−1^ based on sustainable aqueous solutions of enhanced green fluorescent protein and C‐phycocyanin from biological feedstocks. These photonic sensors are presented under the configuration of luminescent solar concentrators widely proposed as a solution to integrate energy‐generating devices in buildings, as windows or façades. The developed mobile sensor is inserted in IoT context through the development of a self‐powered system able to measure, record, and send data to a user‐friendly website.

## Graphical abstract

A sustainable solar optical temperature sensor with thermal sensitivity up to 1.23% °C^−1^ based on aqueous solutions of enhanced Green Fluorescent Protein and C‐phycocyanin is presented. These sensors can be applied as smart windows as the developed sensor is inserted in IoT context through the development of a self‐powered system able to measure and send data to a user‐friendly website.

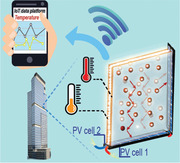

## 1 Introduction

**Figure 1:**
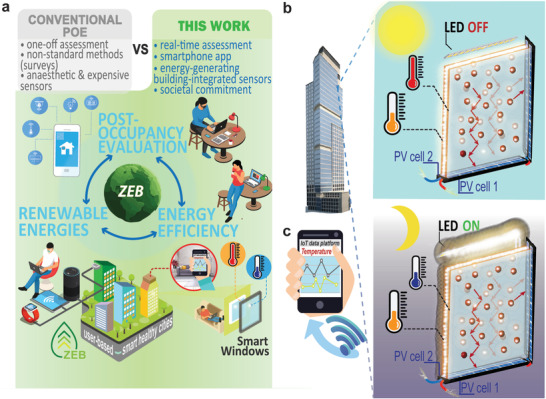
a) Scheme of ZEB implementation fundamentals. b) Schematic representation of the building integrated luminescent solar concentrators (LSCs) as double‐glazed windows attached to Si photovoltaic (PV) cells with intrinsic ability to monitor temperature. LSCs consist of planar waveguides doped or coated with emissive materials that absorb sunlight and re‐emit it at distinct wavelengths. The emitted light is guided toward PV cells coupled to the edges and converted into electricity. The red arrows inside the LSC indicate the total internal reflection of the emitted light. c) Scheme of data transmission to a user‐friendly app/website.

The building sector is among the world's largest energy consumers,^[^ ^1^ ^]^ leading to an intensification in the promotion of net‐zero emission building, often mentioned as Zero Energy Buildings (ZEBs).^[^ ^2^ ^]^ The net‐zero emission buildings require three fundamental conditions (**Figure** **1**a): i) increase energy efficiency, ii) use renewable energies, and iii) implement post‐occupancy evaluation (POE).^[^ ^3^ ^]^ Items (i) and (ii) are mainly addressed by selecting efficient devices and integrating renewable energy sources. Nevertheless, more efficient energy management strategies are required, which can be achieved by the knowledge of population habits related to energy consumption, making POE a key step. Heating is responsible for ≈60% of global CO_2_ emissions^[^ ^1^ ^]^ and thus, thermal comfort is one of the most evaluated parameters in POE protocols^[^ ^4^ ^]^ assessed through surveys and measurements using sensors^[^ ^5^ ^]^ requiring electrical energy. Moreover, those sensors are unable to provide continuous information, simply providing a low sampling rate or unique measurements.

Windows, which account for a significant part of heat gain/loss, can be transformed into energy‐harvesting/generating units through the luminescent solar concentrator (LSC) concept (Figure 1b). LSCs are also an option for on‐site electrical power generation that is environmentally beneficial by avoiding the waste of energy that is lost in transmission and improving energy supply reliability.^[^ ^6^ ^]^ The possibility of using natural renewable materials for LSC applications has been pointed out,^[^ ^7^, ^8^, ^9^, ^10^, ^11^ ^]^ in line with the crescent demand for natural products to be applied in other sectors of activity (e.g., pharmaceutical and biomedicine applications). This is justified by the combined use of bio‐based compounds to increase human health and decrease environmental concerns, considering the use of natural sources instead of synthetic alternatives.^[^ ^12^, ^13^ ^]^ This trend is even more reinforced due to the actual concerns about climate change, but also the new policies to reduce the environmental impact of industrial processes and products,^[^ ^14^ ^]^ particularly when integrated into a smart and low‐waste chain of different products or within a circular economy approach.^[^ ^12^, ^13^, ^15^, ^16^ ^]^

A recent example of bio‐based materials for energy conversion is the enhanced green fluorescent protein (eGFP)^[^ ^11^, ^17^ ^]^ characterized by high thermal, chemical (alkaline pH, detergents, organic salts, proteases), and mechanical (up to 600 MPa) stabilities.^[^ ^18^, ^19^ ^]^ From the optical point of view, eGFP displays emission in the green spectral region, high emission quantum yield (≈0.50), and photostability,^[^ ^11^, ^17^ ^]^ as well as temperature‐dependent optical properties.^[^ ^17^ ^]^ Nonetheless, the emission lies on the green spectral region with a small overlap with Si photovoltaic (PV) cells’ maximum absorbance. C‐phycocyanin (PC), a photosynthetic pigment found mainly in cyanobacteria (renewable and sustainable feedstock)^[^ ^14^ ^]^ that can be extracted with high efficiency^[^ ^20^, ^21^ ^]^ and stability^[^ ^22^, ^23^, ^24^ ^]^ may also be considered for LSCs. PC is characterized by emission in the red spectral region^[^ ^25^ ^]^ closer the maximum absorption of Si PV cells. The concomitant individual contribution of both proteins is beneficial as they present complementary absorption, allowing the application of the concept of tandem LSC formed by two adjacent window panels (LSCs as double‐glazed windows, Figure 1b).

In parallel with improved performance and sustainability, new features must be added to LSC toward sensing to be active in climate change‐related actions and substantial long‐term benefits for society concerning energy consumption habits. The general idea lies in upgrading the concept of LSCs to behave as sunlight‐powered optical temperature sensors. Luminescence is a noninvasive spectroscopic method for temperature measurement based on the thermal dependence of the phosphor emission combining high relative thermal sensitivity (*S*_r _> 1% K^−1^) and spatial resolution (10^−6^ m) with short acquisition times (<10^−3^ s).^[^ ^26^, ^27^, ^28^ ^]^ Very recently, the concept of luminescence thermometry was pushed to mobile optical sensing mediated by smartphones establishing an intriguing strategy to enable large‐scale sensing.^[^ ^29^, ^30^, ^31^ ^]^ Going beyond the state of the art, we propose novel thermometric parameters that include PV cells and smartphones. The thermal‐dependent luminescent properties of emissive materials applied in the LSC will directly impact the number and energy of the photons reaching the PV cells and thus, will determine their electrical output. To keep the system running overnight or during sunlight intermittence periods, indoor lighting can be used to activate artificial PVs (Figure 1b), using an approach similar to that already reported to efficiently pump solid‐state lasers.^[^ ^32^ ^]^ As an added benefit, we will demonstrate the use of the excitation spectra to monitor temperature.

In this pioneer work, a sustainable solar optical temperature sensor based on bio‐based fluorescent proteins was successfully applied using eGFP‐ and PC‐based aqueous solutions for building integration. The thermometric parameter lies on the coupled PV cell open‐circuit voltage, showing a relative thermal sensitivity up to 1.23% °C^−1^. The electrical power delivered by the coupled PV cells under solar radiation is enough to power a small circuit able to read voltage values, convert it to temperature and send real‐time data through Wi‐Fi to a smartphone app or website, bridging these sensors to the Internet of Things (IoT) without increasing the overall energy consumption of the building (Figure 1c). The prototype lies on an integrated device that harvests solar energy and provides continuous measurement of the temperature using bio‐based fluorescent proteins integrated into tandem LSC. This unique combination of sustainable smart photonic windows, optical sensing, and IoT is on the way to contribute to the future design of ZEBs able to enhance energy generation from renewables, increase energy efficiency and management, and shape POE with the users.

## 2 Results and Discussion

### 2.1 Temperature‐Dependent Optical Characterization

**Figure 2:**
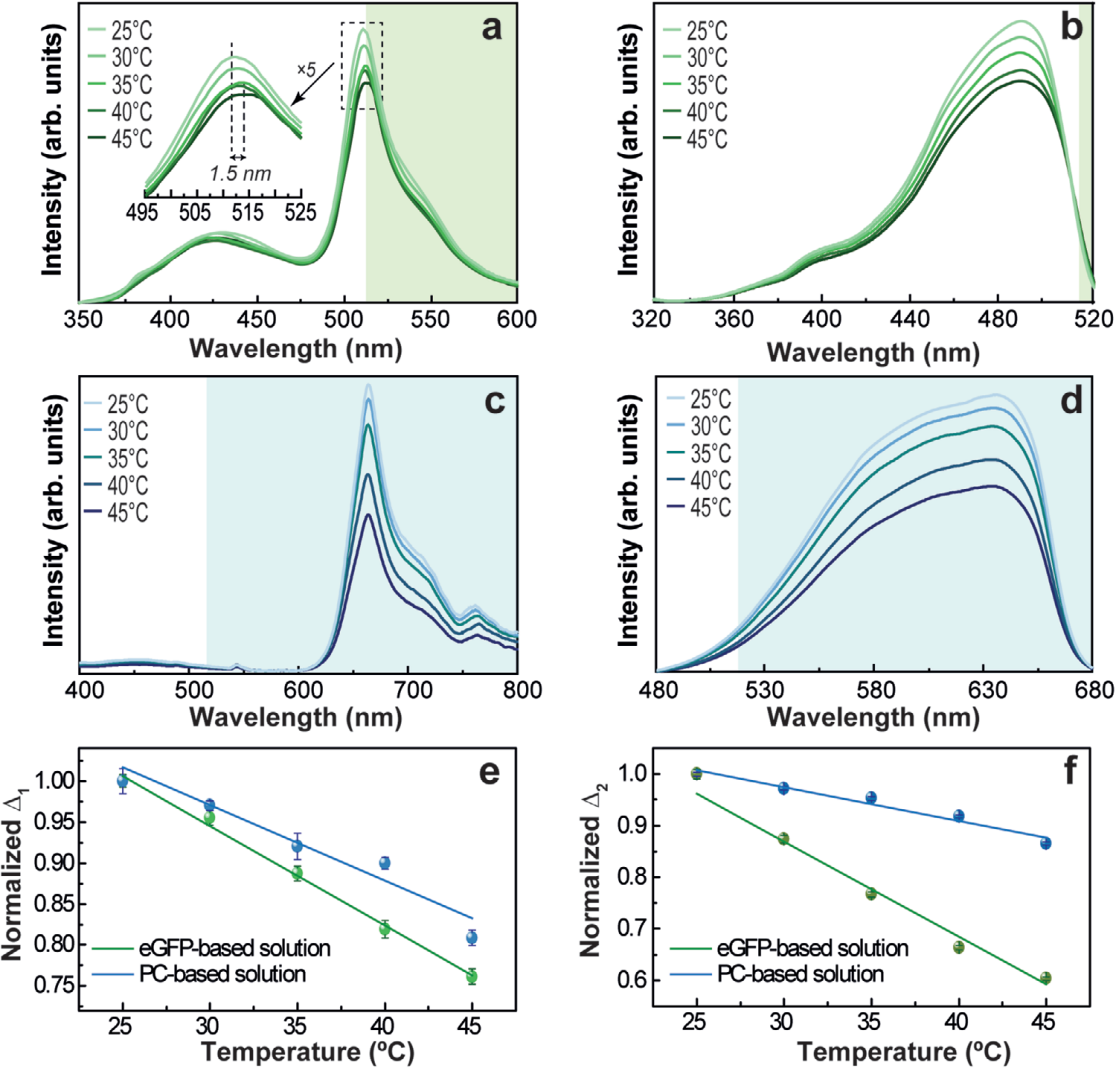
Temperature‐dependent a,c) emission excited at 315 and 380 nm and b,d) excitation spectra monitored at 545 and 715 nm of a,b) eGFP and c,d) PC. The normalized thermometric parameters *∆_i_* are obtained as ratios of the integrated areas in the e) emission and f) excitation spectra; the lines are the best linear fit (*r* ^2 ^> 0.9).

**Figure** **2** shows the photoluminescence features of aqueous solutions of eGFP and PC for selected temperature values. The emission spectra of eGFP are dominated by a band ≈510 nm and a shoulder at 540 nm arising from the I and B excited states,^[^ ^11^ ^]^ respectively. A supplementary emission at 450 nm is due to the relaxation to the ground state of the protonated form (A band).^[^ ^33^ ^]^ For the PC‐based aqueous solution, a red emission centered ≈665 nm is observed.^[^ ^34^ ^]^ The excitation spectra were monitored at the emission maximum intensity. The spectra of eGFP show two components in the blue region ascribed to the A (400 nm) and B (480 nm) bands. ^[^ ^35^, ^36^ ^]^ The spectra of PC are formed by a main PC‐related component (≈660 nm) and the contribution of R‐Phycoerythrin protein excited states in the green spectral region (≈555 nm)^[^ ^25^ ^]^ (Figure 2d). As the temperature is varied, the emission and excitation spectra of eGFP and PC reveal a decrease in the emission intensity (Figure 2). Additionally, a redshift of the emission spectra with the temperature was observed for the eGFP (inset Figure 2a), which is attributed to a charge density transfer occurring between the close neighbors oxygen atoms upon chromophore solvent exposure.^[^ ^17^, ^37^ ^]^

As designed, the PC‐ and eGFP‐based solutions have complementary light‐harvesting ability (Figure S1a, Supporting Information) with the eGFP‐based solution displaying absorption in the 300–500 nm range and the PC‐based solution in the 500–700 nm range with molar extinction coefficient (*ε*) values up to 10^4^ m ^−1^ cm^−1^ (PC) and ≈8 × 10^4^ m ^−1^ cm^−1^ (eGFP). This optical feature will translate into complementary sunlight harvesting ability of the aqueous solutions in which the eGFP lower light harvesting in the red spectral region is raised by the PC contribution, keeping a nearly constant sunlight harvesting ability. The maximum absolute emission quantum yield (*q)* values found are 0.51 ± 0.01 and 0.31 ± 0.03 for the eGFP‐based sample (488 nm excitation) and for the PC‐based aqueous solutions (585 nm excitation), respectively (Table S1, Supporting Information), ensuring efficient sun power conversion, as detailed next.

The light emission efficiency and light harvesting ability can be related by the brightness *B *= *q *× *ε*.^[^ ^38^ ^]^ The *B* values for eGFP and PC solutions are ≈0.5 × 10^4^ and ≈2.5 × 10^4^ m ^−1^ cm^−1^, respectively, which are similar to those reported for orange/red‐emitting organic dyes that are known for their light‐harvesting ability^[^ ^39^ ^]^ and to those previously reported for eGFP‐based materials.^[^ ^11^, ^40^ ^]^ By the estimation of the overlap integral *O* between the absorption spectra and the solar irradiation (AM1.5G), it is found that the eGFP‐ and PC‐based aqueous solutions have the potential to absorb ≈8% (300–700 nm) and ≈3% (500–600 nm) of the solar photon flux on the Earth (4.3 × 10^21^ photons s^−1^ m^−2^),^[^ ^41^ ^]^ respectively (Figure S1b, Supporting Information), while keeping transparency.

Based on the dependence of the photoluminescence on the temperature, we propose the definition of two ratiometric thermometric parameters^[^ ^27^, ^28^, ^29^, ^31^ ^]^ based on the simultaneous use of the emission (Δ_1_ ) and excitation (Δ_2_) spectra, providing dual temperature sensing.

(1) $$\Delta_{1}\;\equiv \frac{S_{A}}{S_{1}}$$

and

(2) $$\Delta_{2}\;\equiv \frac{S_{B}}{S_{2}}$$

where *S_i_* is the total integrated area of the emission (*i *= 1) and excitation (*i *= 2) spectra and *S_A_* and *S_B_* stand for the partial spectral integrated intensity, respectively (shadowed areas in Figure 2; Table S2, Supporting Information). The ratiometric thermometric parameters are independent of sun irradiance fluctuations during a diurnal cycle and reveal a linear dependence on the temperature (Table 1). This is one of the few examples, where the excitation spectra are used to infer temperature being termed as excited state absorption.^[^ ^42^, ^43^ ^]^The figures of merit to evaluate the performance of the thermometers are the relative sensitivity, *S_r_*,^[^ ^44^ ^]^ and the temperature uncertainty, *δT*, defined as:^[^ ^28^ ^]^

(3) $$S_{r}=\frac{1}{\Delta }\;\left|\frac{\partial \Delta }{\partial T}\right|$$

and

(4) $$\delta T\;=\frac{1}{S_{r}}\;\frac{\delta \Delta }{\Delta }\;$$

where *δ*Δ is the uncertainty in the determination of the thermometric parameter Δ. The large *S_r_* values are among the best values found in literature for molecular thermometers^[^ ^29^ ^]^ and the *δT* values are typically below 1 °C (Table 1; Figure S2c,d, Supporting Information) granting the possibility to accurately sense temperature. Moreover, the repeatability tests were performed by measuring the Δ_1_ variation during five sequential cycles between 20 and 35 °C (Figure S3, Supporting Information), showing a repeatability >99% (Supporting Information for details). We note that although ***S***_***r***_ is commonly used as a figure of merit to compare different thermometers, it depends on experimental conditions (e.g., emission spectra resolution) and on the sample characteristics, such as concentration and media.^[^ ^28^ ^]^ Nonetheless, we note that the values here reported are larger than that reported for a thermometer based on eGFP (0.23% °C^−1^).^[^ ^17^ ^]^

**Table 1:** Calibration curve slope (°C^−1^), thermal sensitivity (%°C^−1^) at 25 °C and temperature uncertainty (°C) of the distinct thermometric parameters ∆_1_ – ∆_4_

	Slope [°C^−1^]		*S_r_* [% °C^−1^]		*δT* [°C]	
	eGFP	PC	eGFP	PC	eGFP	PC
∆_1_	−0.0123 ± 0.0005	−0.009 ± 0.001	1.13 ± 0.01	0.91 ± 0.02	0.9	1.3
∆_2_	−0.020 ± 0.001	−0.0065 ± 0.0007	2.00 ± 0.03	0.65 ± 0.01	0.4	0.6
∆_3_	−0.0039 ± 0.0003	−0.0068 ± 0.0003	0.41 ± 0.01	0.72 ± 0.01	0.4	0.1
∆_4_	−0.012 ± 0.004	‐	1.23 ± 0.03	‐	0.02	‐

The combination of these intriguing figures of merit for the thermometric parameter with the optical features (transparency, sunlight harvesting, and down‐shifting) grant both the eGFP or PC solutions as ideal candidates for sustainable temperature sensing. Nonetheless, although the use of a spectrometer is reasonable under laboratory conditions (Δ_1_ and Δ_2_), it is inappropriate if real application is envisaged. With the goal of achieving a temperature sensor to be applied in buildings, as a step forward for the popularization of luminescence thermometry in real‐world applications, we propose a novel thermometric parameter based on the electrical output of PV cells coupled to LSC.

### 2.2 Optical Temperature Sensor Based on LSCs

**Figure 3:**
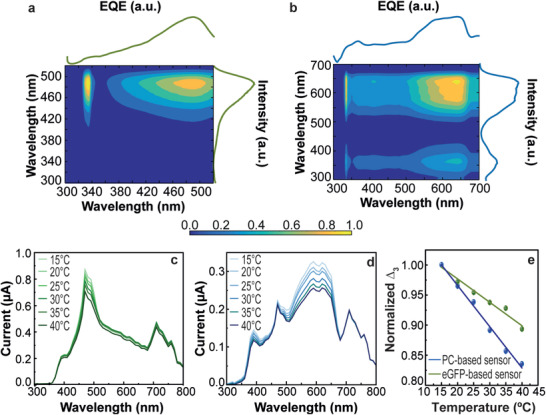
Cross correlation between the EQE of the PV cell coupled to the a) eGFP‐ or b) PC‐based sensors and the excitation spectrum of the correspondent solution. Temperature‐dependent short‐circuit current (*I_sc_)* of c) eGFP‐ or d) PC‐based optical sensors under solar simulator irradiation. e) Temperature calibration curves with the thermometric parameter measured through the *I_sc_* (the lines are the best linear fit with *r* ^2^ > 0.97).

The luminescent solutions were used to fill a glass container emulating a window and fabricate optical temperature sensors (Figure S5a,b, Supporting Information). The photonic sensors were first characterized as LSCs, and the eGFP‐based sensor yielded optical conversion efficiency (*η_opt_*) and power conversion efficiency (PCE) values (Supporting Information for details) of 3.3 ± 0.1% and 0.12 ± 0.01%, respectively. For the case of PC‐based sensor, the maximum *η_opt_* and PCE values were 2.65 ± 0.03% and 0.21 ± 0.01%, respectively. As EQE measurements are the indicators of the number of incident photons of a specific wavelength arriving at the PV cell, an increase in EQE, while illuminating the sensors with eGFP or PC absorption wavelengths indicates that the light arriving at the PV cell has a strong contribution of eGFP or PC converted photons (solar photons absorbed by the luminophores and reemitted). For both cases, EQE measurements showed good correlation with the absorption–excitation spectra of the aqueous solutions, with maximum values of ≈7.6% and ≈2.5% for the eGFP‐ and PC‐based LSCs, respectively (**Figure** **3**a,b).

As the photoluminescence spectra of eGFP and PC are temperature dependent (Figure 2), the optical and electrical performances of the sensors will also vary as function of the temperature under solar simulator irradiation (Figure 3c,d; Figure S5c–e, Supporting Information). It is observed that the short‐circuit current (*I*_sc_) decreases as the temperature elevates enabling the definition of a new thermometric parameter (Δ_3_) based on the PV cell performance:

(5) $$\Delta_{3}\;\equiv \frac{I_{\mathrm{sc}1}}{I_{\mathrm{sc}}}$$

where ***I***_***sc***_ and ***I***_***sc*** 1_ are the integrated short circuit electrical current values (in the spectral ranges selected for Δ_1_, Table S2, Supporting Information). For both samples, the thermometric parameter Δ_3_ also follows a linear dependence with temperature (Figure 3e and Table 1). The figures of merit are analogous to those found for Δ_1_, revealing that the PV thermometric parameter (Δ_3_) is able to sense temperature inside buildings, with *S_r_* values up to 0.73% °C^−1^ for (Figure S6a,b, Supporting Information) and *δT* below 0.4 °C (Table 1; Figure S6c,d, Supporting Information).

**Figure 4:**
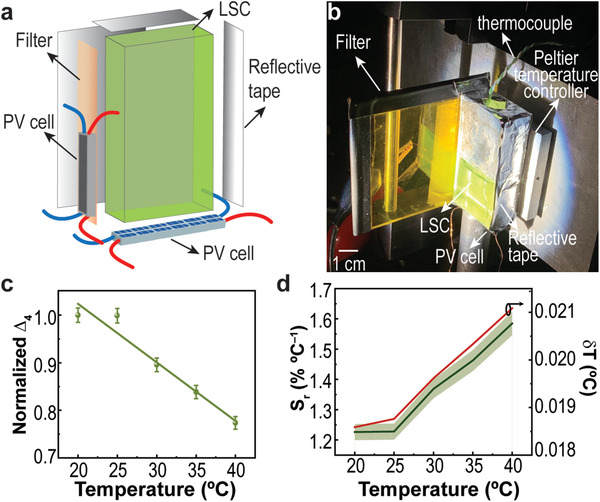
a) Scheme and b) photograph of the setup used for LSC characterization for temperature assessment. The filter used was a 515 nm longpass. c) Temperature calibration curves with the thermometric parameter measured through the delivered *V*_*oc*_ of the LSC coupled PV cells (the line is the best linear fit with *r* ^2 ^> 0.95). d) Relative thermal sensitivity S_r_ calculated using Equation (3) and temperature uncertainty *δT* calculated using Equation (4) for ∆_4_.

Further exploring the definition of the PV thermometric parameter Δ_3_, a sensor prototype was developed. The LSC was exposed to AM1.5G radiation and coupled to commercial Si‐based PV cells. For the illustrative case of the eGFP‐based sensor, as a proof‐of‐concept, the electrical performance of the coupled PV cells was analyzed as function of the temperature, and a thermometric parameter based on the open‐circuit voltage (***V***_***oc***_) was implemented:

(6) $$\Delta_{4}\;\equiv \frac{V_{\mathrm{oc}1}}{V_{\mathrm{oc}}}$$

where *V*_oc1_ is the open‐circuit voltage values measured at the PV cell coupled to the sensor, when coupled to a 515 nm longpass filter (**Figure** **4**a,b). Similar to the above cases, Δ_4_ follows a linear dependence with temperature (Figure 4c,d) with *S_r _* = 1.23% °C^−1^ and *δ*T = 0.02 °C (Table 1).

**Figure 5:**
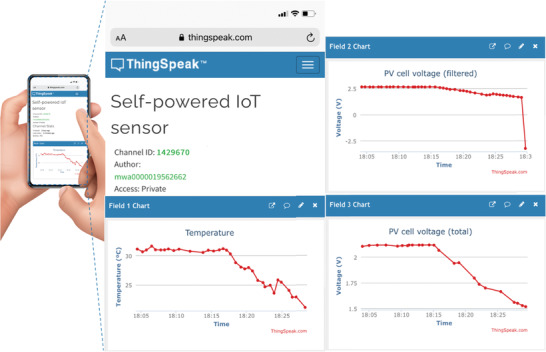
Mobile phone print screen images of the ThingSpeak platform accessed by mobile phone upon temperature measurements with the sensor self‐powered prototype.

After establishing the concept of an optical temperature sensor based on sustainable molecules using the LSC configuration, a practical example for applicability was performed through the development of an IoT system implemented using a programmable board to measure the *V*_oc_ and *V*_oc1_ values (Figure S7, Supporting Information). The voltage values and the estimated temperature are sent by Wi‐Fi to an IoT analytics platform service allowing to aggregate, visualize, and analyze real‐time data streams (**Figure** **5**; Video S1, Supporting Information). The overall sensor electrical output is enough to use part of the PV cells generated electrical current for the measurements and the remaining current being used to power the IoT system, constituting a self‐powered system arising from the fact that the whole sensing and IoT device (in which a battery is included) is powered by the LSC, with no additional need for a power supply. The important issue is to ensure that the average energy consumption (measured over a large period) is less than the energy produced (over the time), which is true in this case. These sensors present the additional advantages of being photostable and the fact of being based on aqueous solutions confers them easy processing and, if necessary, replacement. Moreover, through the collection of temperature data in real‐time through IoT, the involvement of people in today‐decisions related to energy consumption is fostered, making a huge step toward smart and healthy cities. This work states the potential of natural‐based optically active centers to be applied in smart‐windows with inherent sensing abilities.

## 3 Conclusion

A proof‐of‐concept for a solar optical thermal sensor based on the optical properties of natural‐based LSCs with thermal dependent features was reported.

From the concept of LSC a fully functional photonic sensor device operating in the IoT field was demonstrated. This self‐powered system was developed to measure the *V_oc_* values, estimate temperature, and send through Wi‐Fi to a platform service that allows to visualize real‐time data. The obtained information can be used to optimize heating–cooling processes of buildings, with the ultimate purpose of reducing the energy consumption. The thermometric parameter based on the coupled PV cell electrical performance showed a high relative thermal sensitivity (*S_r _* = 1.23% °C^−1^) able to ensure the real temperature sensing. As LSCs are mainly projected to be included in building windows or façades, this work envisages opening the possibility to structures with intrinsic sensing ability. These results validate the potential development of natural‐based LSCs for future energy‐efficient buildings and smart cities, making use of sustainable resources.

## 4 Experimental Section

### Chemical Compounds

The sodium phosphate buffer was prepared using di‐sodium hydrogen phosphate anhydrous (Na_2_HPO_4_, purity 99.0%) and sodium phosphate monobasic (NaH_2_PO_4_, purity 99–100.5%) both acquired at Panreac. Ammonium sulfate (purity 99%) and sodium azide (purity 99%) were purchased from Panreac and Acros Organics, respectively.

### Proteins Extraction

The production of eGFP was performed following the standard procedures described in literature.^[^ ^11^, ^45^ ^]^ An eGFP solution with molecular weight between 25 and 30 kDa was obtained for further use.^[^ ^46^ ^]^ For the PC production and extraction, non‐axenic cultures of the cyanobacteria *Anabaena cylindrica* were established in 5 L schott flasks containing sterilized MBL—Woods Hole culture medium,^[^ ^47^ ^]^ in an incubation chamber at 20 ± 2 °C, with 16 h light(*L*):8 h dark(*D*) photoperiod using 23 00lx provided by cool white fluorescent tubes. After 13 days in culture, the biomass was harvested and concentrated through centrifugation (4 °C; 4111 × *g* for 5 min; Eppendorf 5810 R) for further extraction of PC. The production of the PC solution was performed following standard procedures described in literature.^[^ ^48^ ^]^ As several works have reported light, temperature, and pH as the most relevant parameters disturbing the PC stability,^[^ ^49^ ^]^ which may be attenuated in aqueous solution and controlled by the addition of the sodium azide as a stabilizing agent,^[^ ^50^ ^]^ the fresh biomass was homogenized in sodium phosphate buffer 100 × 10^−3^ m at pH 7, containing 1 × 10^−3^ m of sodium azide to avoid microorganism growth, in a solid–liquid ratio of 1:10. The extraction was performed in an Eppendorf Thermomixer Comfort equipment at 8 × *g* and 30 °C during 50 min. After, the cell suspension was centrifuged at 12 000 × *g* for 10 min in a VVR microstar 17 centrifuge, being the pellet discarded and the supernatant purified. To the liquid fraction (supernatant), 20% (w/v) of ammonium sulfate was added and dissolved, being at 4 °C overnight. The precipitated proteins were recovered by centrifugation at 900 × *g* for 15 min in a VWR microstar 17 centrifuge. The supernatant was discarded, and the pellet was re‐suspended in the same initial volume. A second centrifugation at 9632 × *g* for 5 min was performed. The pellet was discarded, and the supernatant stocked. An ultrafiltration system was performed to the recovered protein fraction after the precipitation step. A 500 µL sample was added in each Amicon Ultra‐0.5 mL Centrifugal Filter Unit 100 K. The sample was centrifuged at 14 000 × *g* during 15 min in a VWR microstar 17 centrifuge. The permeate was discarded and 400 µL of ultrapure water was added to the concentrate and centrifuged in the same conditions, being this last step repeated twice. Lastly, 500 µL of ultrapure water was added to recover the concentrated sample after a centrifugation during 2 min at 1000 × *g*. In the end, a blue extract rich in PC (molecular weight ≈96 kDa)^[^ ^51^ ^]^ was obtained. The concentration for each sample was selected considering the highest performance (molar brightness). The concentration dependence study was performed in the range between 1.4 × 10^−5^ and 5.5 × 10^−5^ m for both eGFP and PC‐based aqueous solutions (Table S1, Supporting Information). The selected concentrations for the sensing application were 5.5 × 10^−5^ m(eGFP)^[^ ^11^ ^]^ and 1.4 × 10^−5^ m (PC).

### Optical Characterization

UV–vis absorption spectra were measured using a Lambda 950 dual‐beam spectrometer (Perkin–Elmer). The absorption spectra were recorded in the temperature range of 15—40 °C with a step of 5 °C. The temperature was increased with a Peltier system (PTP 1, Perkin–Elmer) and recorded using an immersed thermocouple (0.1 °C accuracy, *K*‐type, VWR). The photoluminescence spectra were recorded with a modular double‐grating excitation spectrofluorometer with a TRIAX 320 emission monochromator (Fluorolog‐3, Horiba Scientific) coupled to a R928 Hamamatsu photomultiplier. The emission and excitation spectra were recorded in the temperature range of 25—45 °C with a step of 5 °C. The temperature was increased with a homemade Peltier system (0.1 °C accuracy) and recorded using an immersed thermocouple (0.1 °C accuracy, *K*‐type, VWR). To ensure that the solutions reached the steady‐state temperature, a time interval of 300 s was allowed between consecutive temperature measurements. Emission decay curves (Figure S1c,d, Supporting Information) were recorded at room temperature on a Fluorolog TCSPC spectrofluorometer (Horiba Scientific) coupled to a TBX‐04 photomultiplier tube module (950 V) and a 200 × 10^−9^ s time‐to‐amplitude converter with a delay of 70 × 10^−9^ s. The exciting source was a Horiba/Jobin–Yvon pulsed diode (NanoLED‐390, peak at 388 nm, 1.2 × 10^−9^ s pulse duration, 1 MHz repetition rate, and 150 × 10^−9^ s synchronization delay). The absolute emission quantum yield (*q*) values were measured at room temperature using a system (Quantaurus‐QY Plus C13534, Hamamatsu) with a 150 W xenon lamp coupled to a monochromator for wavelength discrimination, an integrating sphere as the sample chamber, and a multichannel analyzer for signal detection. The method is accurate to within 10%. The eGFP‐based aqueous solution was reported to be stable for several months.^[^ ^11^ ^]^ Both eGFP‐ and PC‐based aqueous solutions kept their optical properties after more than 12 months storage and regular testing.

### Temperature‐Dependent Measurements

Temperature‐dependent fluorescence emission spectra of eGFP and PC solutions were obtained when the solution was irradiated with AM1.5G solar simulator. The detection system to collect the emission spectra used an optical fiber connected to a portable spectrometer (SensLine, AVANTES, slit 100 µm) for real‐time acquisition (integration time of 6 ms and 50 readings). For calibration measurements, the temperature was increased using a Peltier‐based temperature‐controlled cuvette holder (TLC 50, Quantum Northwest) with accuracy of ±0.2 °C, and recorded using an immersed thermocouple (0.1 °C accuracy, *K*‐type, VWR). The temperature dependence of the *I_sc_* delivered by the PV cell coupled to the LSCs was measured in the temperature range of 15—40 °C with a step of 5 °C. To have a calibration curve independent of the power fluctuations of the solar simulator, the Δ_3_ values were normalized to the ones at 15 °C. The temperature was increased with a homemade Peltier system (0.1 °C accuracy) and recorded using an immersed thermocouple (0.1 °C accuracy, *K*‐type, VWR). For the LSC sensor prototype testing, one of the edges was directly coupled to a PV cell and the other edge had a longpass filter, with a transmission edge at 515 nm, between the LSC and the PV cell (Figure 4a,b). The longpass filter was included to discriminate the LSC emitted photons with a wavelength higher 515 nm from the remaining emitted photons. This approach allowed the implementation of a ratiometric thermometer, following the methodologies above mentioned, which was less prone to external influences. In this case, to have a calibration curve independent of the power fluctuations of the solar simulator, the Δ_4_ values were normalized to the ones at 20 °C.

## Conflict of Interest

The authors declare no conflict of interest.

## Supporting information

Supporting Information

## Data Availability

The data that support the findings of this study are available from the corresponding author upon reasonable request.
