# Supplementary material for: Bio‐Based Solar Energy Harvesting for Onsite Mobile Optical Temperature Sensing in Smart Cities
Source: Adv Sci (Weinh). 2022 Mar 28;9(17):2104801. doi: 10.1002/advs.202104801 (PMC9189672; doi:10.1002/advs.202104801)
Supplement: Supplementary file 1 — Supporting Information [file ADVS-9-2104801-s001.pdf]

ADVANCED SCIENCE
Open Access

Supporting Information

for *Adv. Sci.*, DOI 10.1002/advs.202104801

Bio-Based Solar Energy Harvesting for Onsite Mobile Optical Temperature Sensing in Smart Cities

Sandra F.H. Correia, Ana R.N. Bastos, Margarida Martins, Inês P.E. Macário, Telma Veloso, Joana L. Pereira, João A.P. Coutinho, Sónia P.M. Ventura, Paulo S. André and Rute A.S. Ferreira**

Supporting Information

Bio-based solar energy harvesting for onsite mobile optical temperature sensing in smart cities

Sandra F.H. Correia,[¥] Ana R.N. Bastos,[¥] Margarida Martins, Inês P.E. Macário, Telma Veloso, Joana L. Pereira, João A.P. Coutinho, Sónia P.M. Ventura, Paulo S. André and Rute A.S. Ferreira**

Contents

S1. Optical characterization

S2. Thermometric characterization

S3. Repeatability calculation

S4. LSCs performance evaluation parameters

S5. Sun-powered IoT optical temperature sensor

Table S1. Integral overlap (O, photons·s^{-1}·m^{-2}), molar extinction coefficient (ε, M^{-1}·cm^{-1}), absolute emission quantum yield (q) and brightness (B, M^{-1}·cm^{-1}) of the aqueous solutions ([ ], M). The ε, q and B values refer to 488 and 585 nm for eGFP and PC-based solutions, respectively.

Table S2. Spectral regions considered for the estimation of the $\boldsymbol{\phi_f}$ parameter used in Δ_1 and Δ_2.

Figure S1. (a) Absolute absorbance of eGFP and PC aqueous solutions, and (b) integral overlap between the solar photon flux and the absolute absorbance for each one. Room-temperature emission decay curve of (c) eGFP- and (d) PC-based aqueous solutions excited at 388 nm and monitored at 510 and 670 nm, respectively. The solid lines represent the data best fit (R^2 >0.99), using a single-exponential function $I(t) = I_1 e^{-(t-t_0)/\tau_1}$ (t_0 = 15 ns, related with the excitation prompt).

Figure S2. Relative thermal sensitivity S_r calculated using equation (3) for (a) Δ_1 and (b) Δ_2; Temperature uncertainty δT calculated using equation (4) for (c) Δ_1 and (d) Δ_2.

Figure S3. Normalized thermometric parameter Δ_1 recorded in 5 heating-cooling temperature cycles at 20 and 35 °C showing repeatability higher than 99%.

Figure S4. Reflectance curve of the reflective tape used on the LSCs.

Figure S5. Photograph of the sensor based on a glass container filled with (a) eGFP- or (b) PC-based aqueous solutions under AM1.5G illumination. The PV cell is located at the bottom edge of the container. Temperature-dependent emission spectra under the solar simulator of (c) eGFP- and (d) PC-based sensors. (e) Temperature calibration curves with the

thermometric parameter measured through the emission intensity (the line is the best linear fit with $r^2 > 0.97$).

Figure S6. Relative thermal sensitivity S_r calculated using equation (3) for (a) Δ_1 and (b) Δ_3; Temperature uncertainty δT calculated using equation (4) for (c) Δ_1 and (d) Δ_3.

Figure S7. V_{oc} and V_{oc1} values of the eGFP-based sensor according to temperature, used to calculate Δ_4.

Figure S8. Schematic representation of the Sun-powered IoT optical temperature sensor. The red lines correspond to the positive terminals, and the black lines correspond to the ground (GND). The grey dots in the ESP8266 Thing board correspond to the connections with a FTDI Basic Breakout board to send the code to the ESP8266 through a serial communication.

Figure S9. Frame of the video depicting the Sun-powered IoT optical temperature sensor. In the video, it is possible to view the system working autonomously. The temperature of the LSC was varied using an external Peltier system. A new data point is acquired every 15 seconds and, thus, the video was accelerated. The full video is found as Supporting Information.

S1. Optical characterization

The absorption spectra resemble the excitation ones. The estimated molar extinction coefficient values (ε) values are lower than those previously reported for another eGFP-based solution prepared by a distinct methodology[1] and for a low concentrated solution (~10^{-6} M) of PC in phosphate buffers.[2,3] We notice that the evidence that the extraction methodology and the effect of the concentration on the optical absorption is a relevant aspect but lies beyond the scope of the present work. Here, the optical parameters were optimised to ensure enough sunlight harvesting while keeping transparency.

At room-temperature, the emission decay curves of the eGFP and PC-based aqueous solutions were also measured, being well described by a single exponential function (Figure S1c,d), yielding lifetime values of τ_1=2.91±0.02 ns for the eGFP case, similar to those previously reported (~3 ns). For the PC-based solutions, the lifetime values were found to be τ_1=2.54±0.01 ns, which are above the ones reported for PC-based samples around 1.5 ns.[4-6]

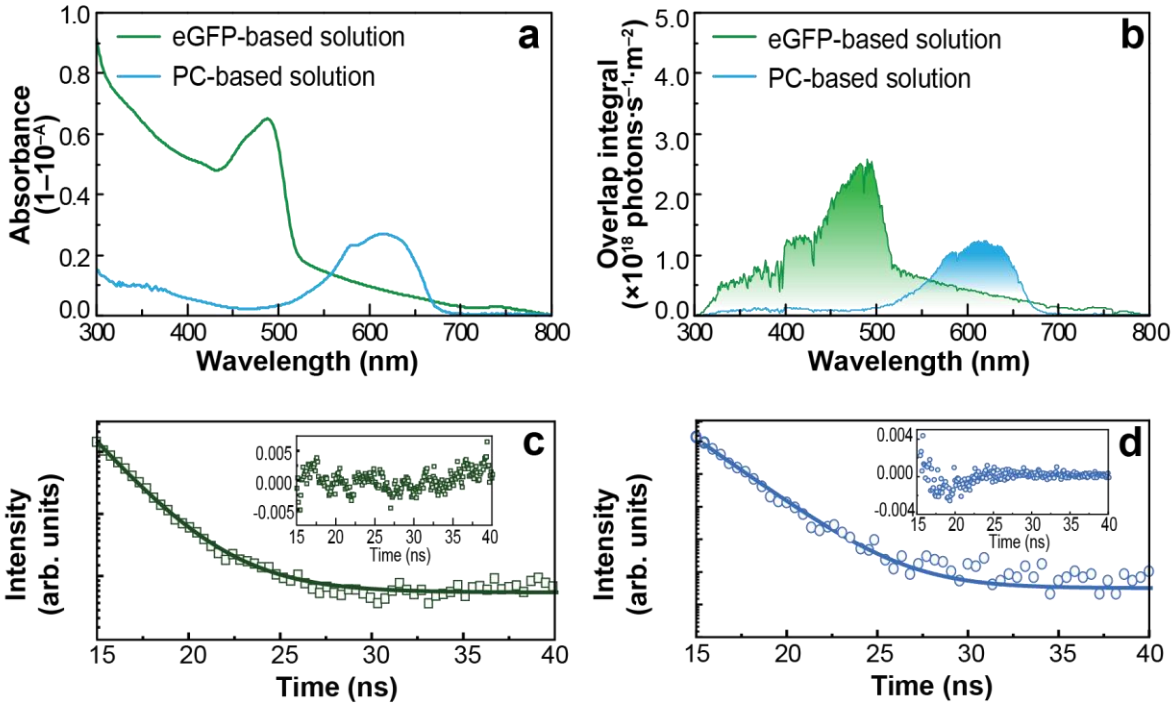

Figure S1. (a) Absolute absorbance of eGFP and PC aqueous solutions, and (b) integral overlap between the solar photon flux and the absolute absorbance for each one. Room-temperature emission decay curve of (c) eGFP- and (d) PC-based aqueous solutions excited at 388 nm and monitored at 510 and 670 nm, respectively. The solid lines represent the data best fit (R^2 >0.99), using a single-exponential function $I(t) = I_1 e^{-(t-t_0)/\tau_1}$ (t_0 = 15 ns, related with the excitation prompt).

The absolute emission quantum yield (*q)* values (Table S1) are lower than those previously reported in the literature which are 0.80 for eGFP in phosphate buffers for more concentrated samples (~10^{-2} M)[7] and ~0.68*,* found for PC in phosphate buffers for a less concentrated sample (~10^{-6} M).[2,7]

To quantify the ability of the LSCs to absorb the sunlight available for PV conversion, the overlap integral *O* between the eGFP and PC aqueous solutions absorption spectra and the solar irradiation spectra was calculated.[8]

$$O = \int_{\lambda_1}^{\lambda_2} N_{AM1.5G}(\lambda) \times \left(1 - 10^{-A(\lambda)}\right) d\lambda \qquad \text{(S1)}$$

where λ_1 and λ_2 are the limits of the spectral overlap between the absorption spectrum of the solutions and the AM1.5G spectrum, $N_{\mathrm{AM1.5G}}$ is the photon flux of AM1.5G and *A* is the absorbance of the solutions.

Table S1. Integral overlap (O, photons·s^{-1}·m^{-2}), molar extinction coefficient (ε, M^{-1}·cm^{-1}), absolute emission quantum yield (*q*) and brightness (B, M^{-1}·cm^{-1}) of the aqueous solutions ([ ], M). The ε, *q* and B values refer to 488 and 585 nm for eGFP and PC-based solutions, respectively.

**Aqueous solutions**	**[ ] (×10^{-5})**	***O* (×10^{20})**	***ε* (×10^{4})**	***q***	***B* (×10^{4})**
eGFP	1.4	0.9	1.0	0.49±0.05	0.5
	3.5	2.2	0.93	0.52±0.05	0.5
	5.5	3.4	0.99	0.51±0.05	0.5
PC	1.4	1.4	8.13	0.31±0.03	2.5
	3.5	2.9	7.85	0.26±0.03	2.0
	5.5	4.0	7.77	0.26±0.03	2.1

S2. Thermometric characterization

Table S2. Spectral regions considered for the estimation of the S_1 and S_2 parameters used in Δ_1 and Δ_2.

	Spectral region considered [nm]	
	**eGFP**	**PC**
S_A	515-600	515-800
S_B	515-520	515-700

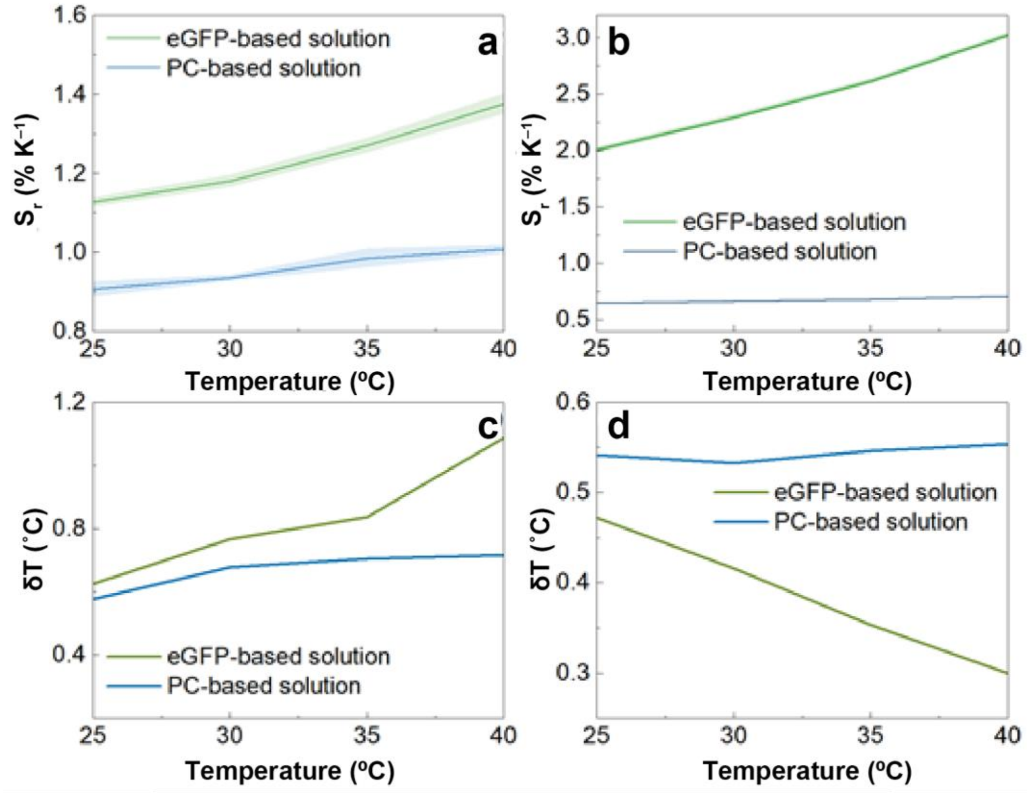

Figure S2. Relative thermal sensitivity, equation (3), for (a) Δ_1 and (b) Δ_2 and temperature uncertainty, equation (4), for (c) Δ_1 and (d) Δ_2.

S3. Repeatability calculation

The repeatability refers to the variation in repeated measurements performed under identical conditions. The repeatability was estimated by cycling the temperature between 20 to 35 °C, ensuring the thermal equilibrium between each measurement. The repeatability of a thermometer's readout upon temperature cycling was then quantified using the expression:

$$R = 1 - \frac{\max(|\Delta_c - \Delta_i|)}{\Delta_c} \tag{S2}$$

where Δ_c is the mean thermometric parameter and Δ_i is the value of each measurement of the thermometric parameter.

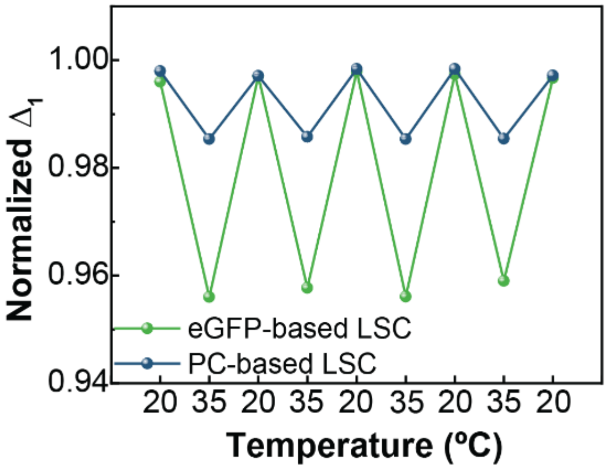

Figure S3. Normalized thermometric parameter Δ_1 recorded in 5 heating-cooling temperature cycles at 20 and 35 °C showing repeatability higher than 99%.

S4. LSCs performance evaluation parameters

The optical conversion efficiency (η_{opt}) was estimated by equation (S3):[9]

$$\eta_{opt}=\frac{P_{out}}{P_{in}}=\frac{I_{SC}^{L}V_{oc}^{L}}{I_{SC}V_{oc}}\frac{A_e}{A_s}\frac{\eta_{solar}}{\eta_{PV}} \tag{S3}$$

where I_{SC}^{L} and V_{oc}^{L} represent the short-circuit current and the open-circuit voltage when the PV device is coupled to the LSC, I_{sc} and V_{oc} are the corresponding values of the PV device exposed directly to the solar radiation, A_s and A_e are the exposed and total edge area respectively, η_{solar} is the efficiency of the PV device relatively to the total solar spectrum and η_{PV} is the efficiency of the PV device at the LSC emission wavelengths. Three measurements were performed for each case, with a found relative error ($\eta_{opt}/\Delta\eta_{opt}$) below 10 %.

The experimental η_{opt} values were determined by illuminating the top surface of the LSCs (2.0×2.0 cm^2) with AM1.5G illumination from a solar simulator. The optical power at the LSC output was estimated using a commercial c-Si PV panel (KXOB22-01X8F, IXYS, composed by 8 cells with 2.4×5.1 mm^2 active area) coupled to one edge of the LSC (2.0×1.0 cm^2), while the remaining edges were covered with reflective tape (Figure S4). The I_{sc} and V_{oc} values were measured using a current source meter device (2400 SourceMeter SMU Instruments, Keithley). All measurements were performed under AM1.5G illumination (1000 $W{\cdot}m^{-2}$) using a 150 W xenon arc lamp, class A, solar simulator (Model 10500, Abet Technologies). The mismatch in the UV spectral region between the AM1.5G solar irradiance and that of the Xe lamp in the solar simulator was taken into consideration following a methodology reported in detail elsewhere.[10] Since the incident radiation from solar simulator induces some heating of the LSCs, the external quantum efficiency (EQE) measurements were performed to the PV cell coupled to the LSCs with controlled temperature conditions and was calculated following equation (S4):

$$EQE(\lambda)=\frac{I_{SC}{\cdot}h{\cdot}c}{P_{in}{\cdot}e{\cdot}\lambda} \tag{S4}$$

where e is the charge of the electron, h is the Planck's constant, c is the speed of light and λ is the wavelength. The solar simulator was coupled to a monochromator (Triax 180, Horiba Scientific). The I_{sc} and P_{in} values were measured using the above-mentioned current sourcemeter and a c-Si calibrated photodiode (FDS1010, Thorlabs), respectively. The power conversion efficiency (PCE) was calculated following equation (S5):

$$PCE=\frac{P_{out}^{el}}{P_{in}}=\frac{I_{SC}^{L}V_{oc}^{L}FF}{A_S\int_{\lambda_1}^{\lambda_2}I_{AM1.5G}(\lambda)d\lambda} \tag{S5}$$

where P_{out}^{el} and FF=0.75 are the PV device output electrical power and fill factor of the PV cell, respectively.

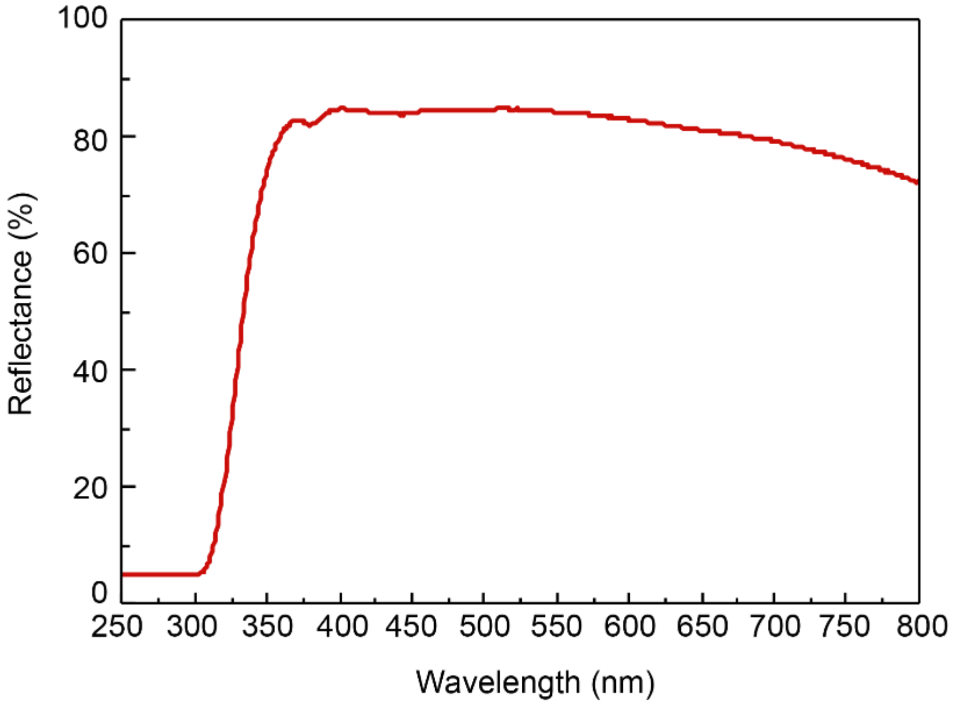

Figure S4. Reflectance curve of the reflective tape used on the LSCs.

S5. Sun-powered IoT optical temperature sensor

As the photoluminescence spectra of eGFP and PC are temperature dependent, the optical and electrical performances of the sensors were also tested as function of the temperature (Figure 3 and Figure S5). Initially, the sensors were exposed to incident radiation from the solar simulator, and the emission spectra were obtained in the temperature range of 15–40 °C with a step of 5 °C (Figure S5c,d). The emission spectra consist in an overlap of the aqueous solutions emission and the solar radiation spectra. As expected by the photoluminescence results depicted in Figure 2, the emission spectra under solar radiation also presented a temperature dependence. Taking these variations into account, the thermometric parameter in this situation is similar to the Δ_1 mentioned in the manuscript in equation (1).

To have a calibration curve independent of the power fluctuations of the solar simulator, the Δ_1 values were normalized to the ones at 15 °C, respectively. For both samples, the thermometric parameter Δ_1 follow a linear dependence with temperature (Figure S5e), with a slope of −0.0019 ± 0.0001 °C^{-1} and −0.00071 ± 0.00002 °C^{-1} for the eGFP- and PC-based sensors, respectively.

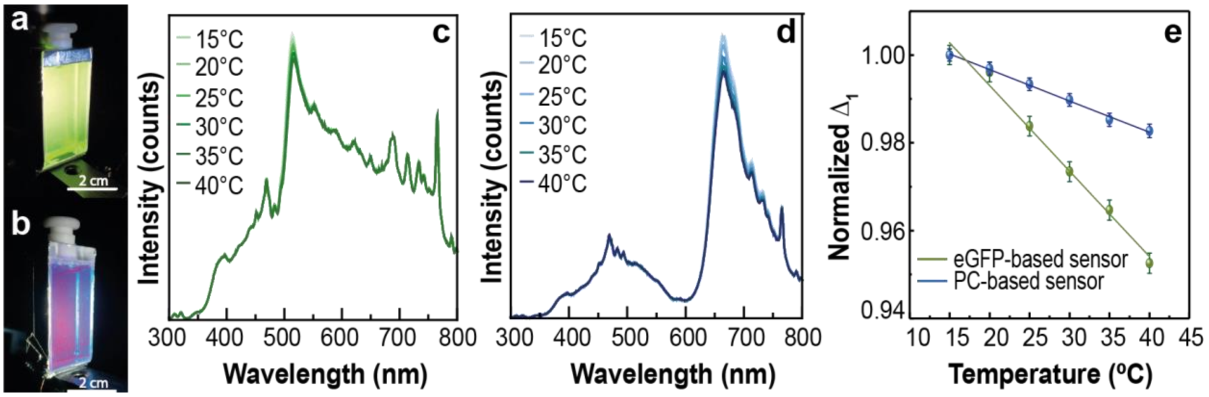

Figure S5. Photograph of the sensor based on a glass container filled with (a) eGFP- or (b) PC-based aqueous solutions under AM1.5G illumination. The PV cell is located at the bottom edge of the container. Temperature-dependent emission spectra under the solar simulator of (c) eGFP- and (d) PC-based sensors. (e) Temperature calibration curves with the thermometric parameter measured through the emission intensity (the line is the best linear fit with $r^2 > 0.97$).

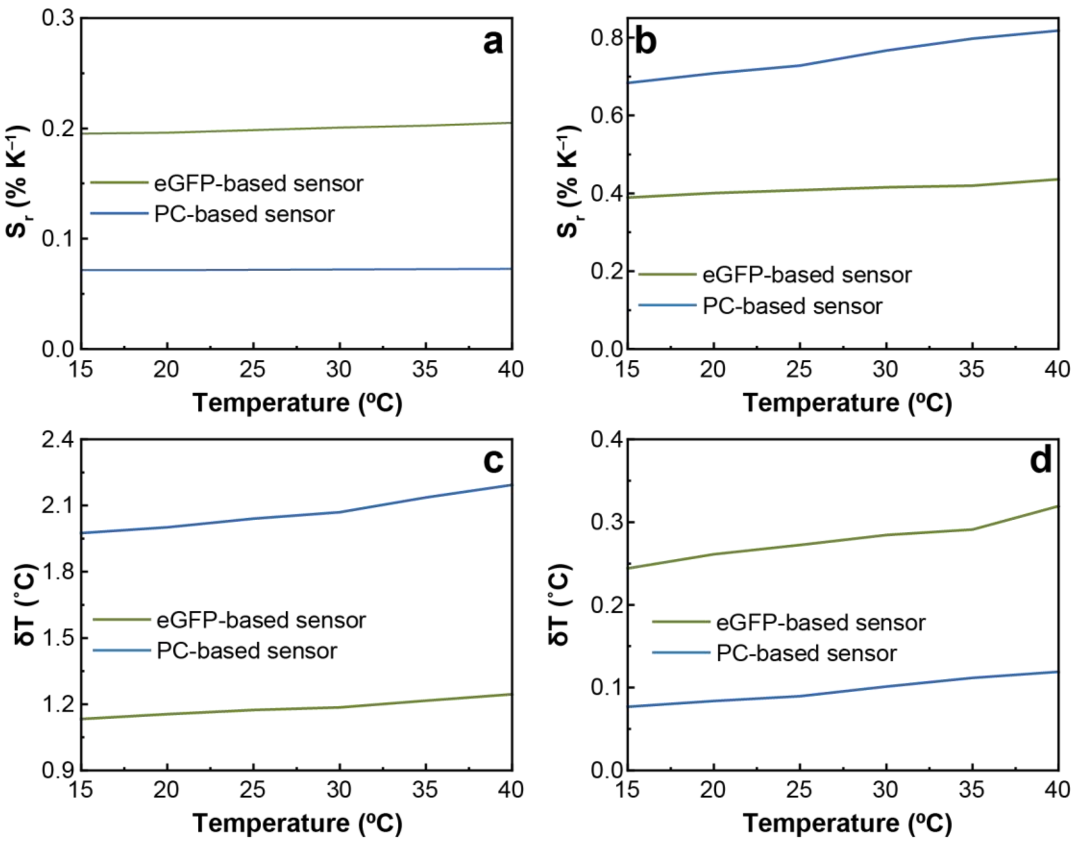

Figure S6. Relative thermal sensitivity S_r, equation (3) for (a) Δ_1 and (b) Δ_3 and temperature uncertainty, equation (4), for (c) Δ_1 and (d) Δ_3.

Temperature-dependent V_{oc} values of the PV cells coupled to the sensors based on eGFP and PC solutions were obtained, upon irradiation with AM1.5G. The temperature dependence of the V_{oc} delivered by the PV cell coupled to the sensors was measured in the temperature range of 20-40 °C with a step of 5 °C. The temperature was increased with a homemade Peltier system (0.1 °C accuracy) and recorded using an immersed thermocouple (0.1 °C accuracy, K-type, VWR). Two different voltage values were obtained, one with a PV cell directly coupled to the sensor (V_{oc}) and another one with PV cell coupled to the sensor with a filter (515 nm longpass) in between (V_{oc1}), Figure S7. The IoT system was implemented in a programmable board (ESP8266 Thing, SparkFun) to measure the V_{oc1} and V_{oc} values, estimate the temperature using the calibration curve (Figure 4c), and send the temperature values in real-time to a data server. The two voltage values were measured using an analog-to-digital converter extender (ADC, ADS1115 16 Bit module with 4 channels) connected to the board using the I2C communication protocol. The ADCs were programmed to measure voltage values between ±512.00 mV with a resolution of 0.25 mV, requiring a minimum current of 70 µA. So, a signal conditioning circuit was implemented (Figure S8), with precision resistors in a voltage divider configuration and ensuring the minimum current for detection

by the ADC, to use part of the PV cell current for the measurements with the ADCs (80 - 130 μA) and the remaining current to power the IoT system (I_1 and I_2, 160 - 260 μA). The voltage values and the estimated temperature are sent by wi-fi to an IoT analytics platform service allowing to aggregate, visualize, and analyse real-time data streams in the cloud. The ThingSpeak™ platform was used, where a channel with three fields was developed, two fields for the voltage values and the last one for the temperature. The values for each field are updated at each 15 seconds.

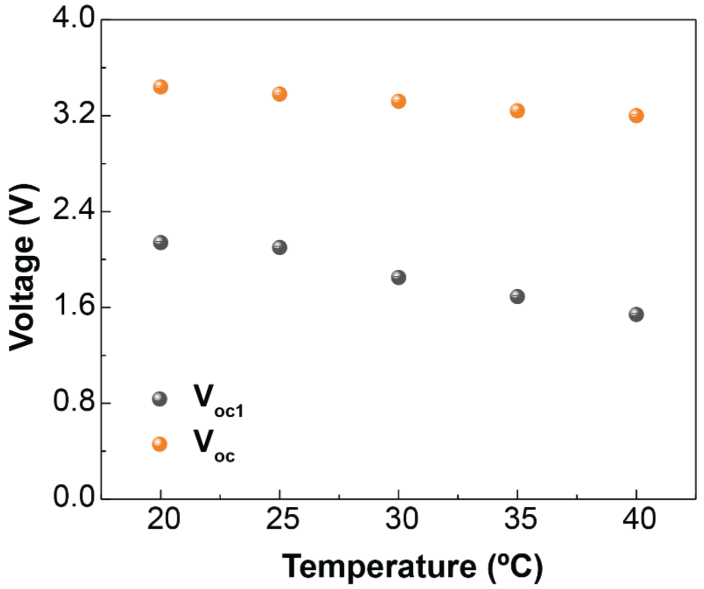

Figure S7. V_{oc} and V_{oc1} values of the eGFP-based sensor according to temperature, used to calculate Δ_4.

To power the ESP8266 board, the I_f and I_t were added by connection of the positive terminals and the negative ones, yielding an output voltage and current of ~90 mV and ~340 μA, respectively. To manage the harvested energy and prevent a battery over-voltage and reverse polarity connection, a battery charger module (TP4056) was used to maintain a constant-current/constant-voltage linear charger in a single cell lithium-ion battery (Figure S8). The rechargeable Lithium battery was connected to the battery charger module which outputs a voltage of ~3.9 V. As the board requires only 3.3 V, a voltage regulator was used to power the ESP8266 board with 3.3 V. As the values are updated within an interval of 15 seconds, between the measurements the ESP8166 is the deep-sleep mode, requiring only ~10 μA. During the transmission by wi-fi, the board required ~135 mA, which is stored in the battery during the deep-sleep regime. We note that although the IoT circuit includes a battery, it is powered by the LSC's electrical output and used for energy storage to cope with possible supply failures and for the periods where the Wi-fi connection requires a high energy demand.

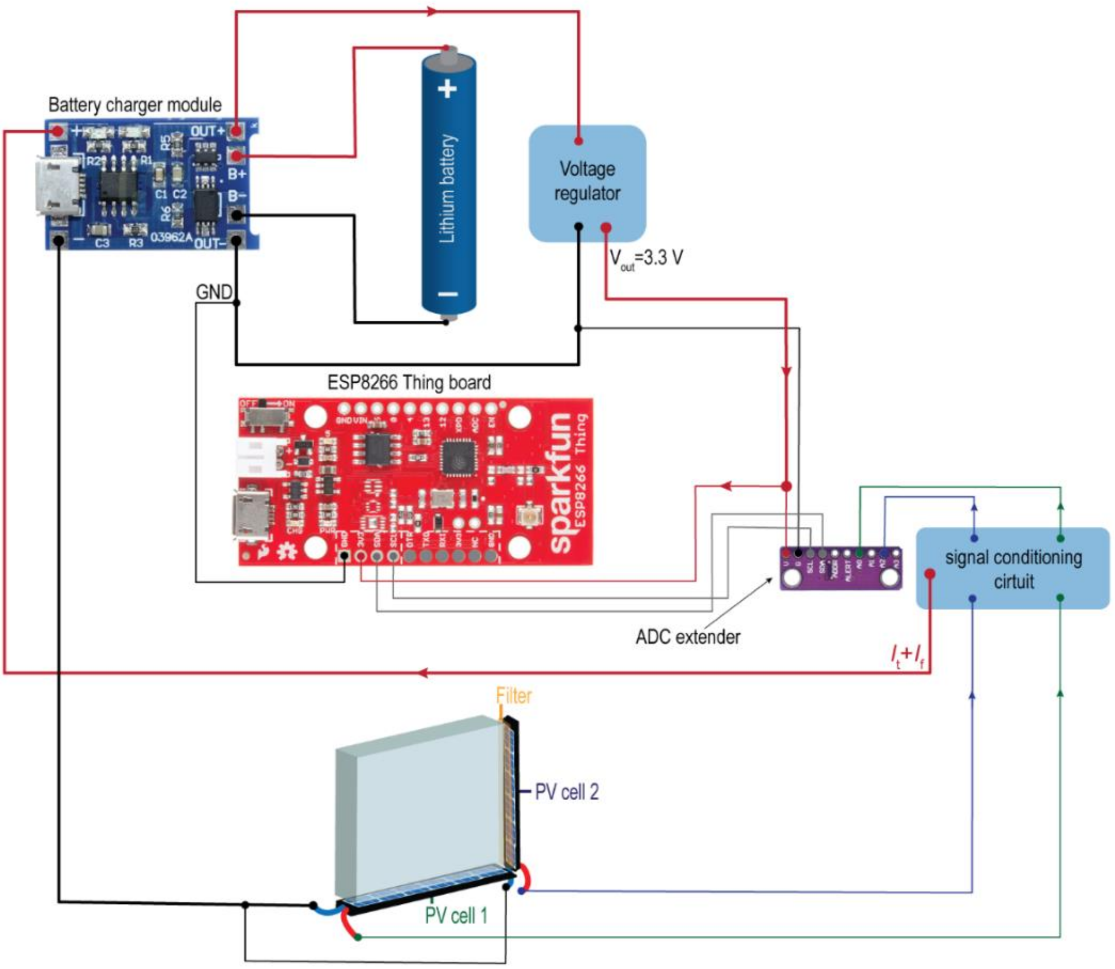

Figure S8. Schematic representation of the Sun-powered IoT optical temperature sensor. The red lines correspond to the positive terminals, and the black lines correspond to the ground (GND). The grey dots in the ESP8266 Thing board correspond to the connections with a FTDI Basic Breakout board to send the code to the ESP8266 through a serial communication.

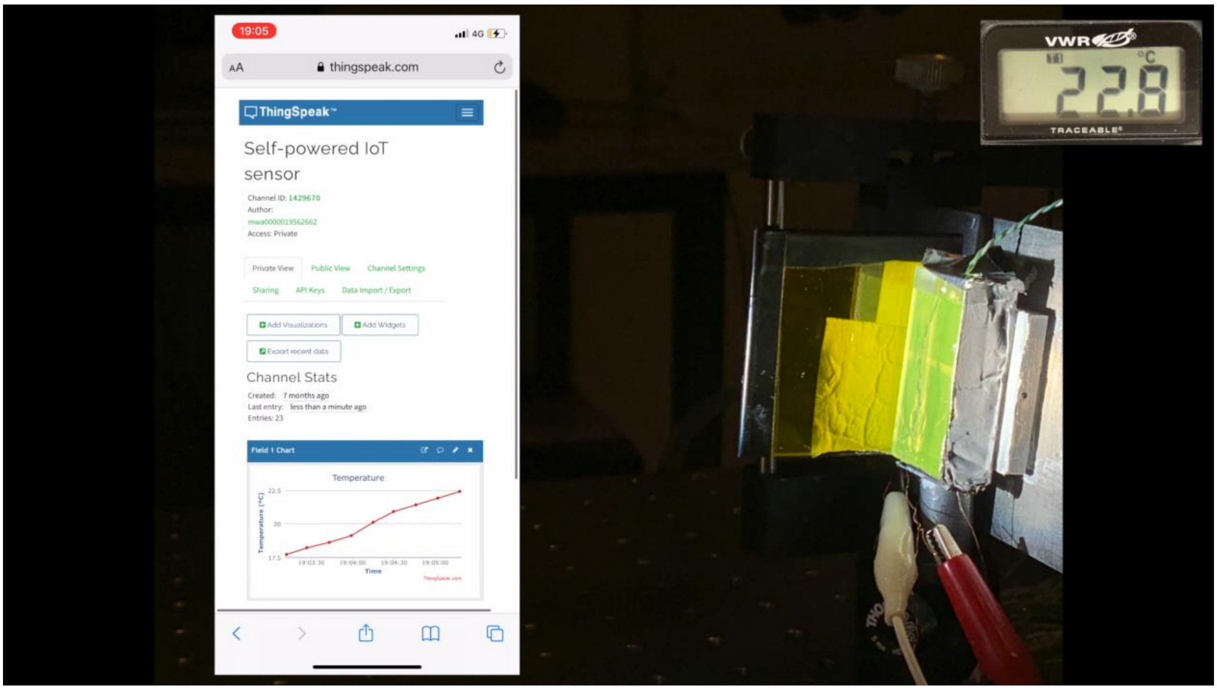

Figure S9. Frame of the video depicting the Sun-powered IoT optical temperature sensor. In the video, it is possible to view the system working autonomously. The temperature of the LSC was varied using an external Peltier system. A new data point is acquired every 15 seconds and, thus, the video was accelerated. The full video is found as Supporting Information.

References

[1] M. Chalfie, *Photochem. Photobiol.*, **1995**, 62, 651.

[2] J. Grabowski, E. Gantt, *Photochem. Photobiol.*, **1978**, 28, 39.

[3] G. T. Hermanson, *Bioconjugate Techniques*, 3rd edition, Academic Press, **2013**.

[4] A. H. Squires, W. E. Moerner, *P. Natl. Acad. Sci. USA*, **2017**, 114, 9779.

[5] M. Seibert, J. S. Connolly, *Photochem. Photobiol.*, **1984**, 40, 267.

[6] S. S. Brody, in *Discoveries in Photosynthesis*, Vol. 20 (Eds: Govindjee, J. T. Beatty, H. Gest, J. F. Allen), Springer, The Netherlands 2005.

[7] H. Morise, O. Shimomura, F. H. Johnson, J. Winant, *Biochemistry*, **1974**, 13, 2656.

[8] R. Rondão, A. R. Frias, S. F. H. Correia, L. S. Fu, V. de Zea Bermudez, P. S. André, R. A. S. Ferreira, L. D. Carlos, *ACS Appl. Mater. Interfaces*, **2017**, 9, 12540.

[9] R. Reisfeld, D. Shamrakov, C. Jorgensen, *Sol. Energ. Mat. Sol. C*, **1994**, 33, 417.

[10] S. F. H. Correia, P. P. Lima, P. S. Andre, R. A. S. Ferreira, L. D. Carlos, *Sol. Energ. Mat. Sol. C.*, **2015**, 138, 51.
